# Expressing the *Thermoanaerobacterium saccharolyticum pforA* in engineered *Clostridium thermocellum* improves ethanol production

**DOI:** 10.1186/s13068-018-1245-2

**Published:** 2018-09-06

**Authors:** Shuen Hon, Evert K. Holwerda, Robert S. Worthen, Marybeth I. Maloney, Liang Tian, Jingxuan Cui, Paul P. Lin, Lee R. Lynd, Daniel G. Olson

**Affiliations:** 1 Thayer School of Engineering, Dartmouth College, 14 Engineering Drive, Hanover, NH 03755 USA; 2 Bioenergy Science Center, Oak Ridge National Laboratories, Oak Ridge, TN 37830 USA; 3 Center for Bioenergy Innovation, Oak Ridge National Laboratories, Oak Ridge, TN 37830 USA; 4 Department of Biological Sciences, Dartmouth College, Hanover, NH 03755 USA; 5 University of California, Los Angeles, Los Angeles, CA 90095 USA

**Keywords:** Consolidated bioprocessing, *Clostridium thermocellum*, *Thermoanaerobacterium saccharolyticum*, Pyruvate ferredoxin oxidoreductase, Ethanol, Isobutanol

## Abstract

**Background:**

*Clostridium thermocellum* has been the subject of multiple metabolic engineering strategies to improve its ability to ferment cellulose to ethanol, with varying degrees of success. For ethanol production in *C. thermocellum*, the conversion of pyruvate to acetyl-CoA is catalyzed primarily by the pyruvate ferredoxin oxidoreductase (PFOR) pathway. *Thermoanaerobacterium saccharolyticum*, which was previously engineered to produce ethanol of high yield (> 80%) and titer (70 g/L), also uses a pyruvate ferredoxin oxidoreductase, *pforA*, for ethanol production.

**Results:**

Here, we introduced the *T. saccharolyticum pforA* and ferredoxin into *C. thermocellum*. The introduction of *pforA* resulted in significant improvements to ethanol yield and titer in *C. thermocellum* grown on 50 g/L of cellobiose, but only when four other *T. saccharolyticum* genes (*adhA*, *nfnA*, *nfnB*, and *adhE*^*G544D*^) were also present. *T. saccharolyticum* ferredoxin did not have any observable impact on ethanol production. The improvement to ethanol production was sustained even when all annotated native *C. thermocellum pfor* genes were deleted. On high cellulose concentrations, the maximum ethanol titer achieved by this engineered *C. thermocellum* strain from 100 g/L Avicel was 25 g/L, compared to 22 g/L for the reference strain, LL1319 (*adhA*(*Tsc*)-*nfnAB*(*Tsc*)-*adhE*^G544D^ (*Tsc*)) under similar conditions. In addition, we also observed that deletion of the *C. thermocellum pfor4* results in a significant decrease in isobutanol production.

**Conclusions:**

Here, we demonstrate that the *pforA* gene can improve ethanol production in *C. thermocellum* as part of the *T. saccharolyticum* pyruvate-to-ethanol pathway. In our previous strain, high-yield (~ 75% of theoretical) ethanol production could be achieved with at most 20 g/L substrate. In this strain, high-yield ethanol production can be achieved up to 50 g/L substrate. Furthermore, the introduction of *pforA* increased the maximum titer by 14%.

**Electronic supplementary material:**

The online version of this article (10.1186/s13068-018-1245-2) contains supplementary material, which is available to authorized users.

## Background

*Clostridium thermocellum* is a promising candidate organism for the consolidated bioprocessing (CBP) of lignocellulosic biomass into biofuels, such as ethanol [1]. Metabolic engineering of *C. thermocellum* has improved ethanol yields and titers; however, further improvements are needed for commercial viability [1–3]. The metabolic engineering strategies pursued in *C. thermocellum* have encompassed restricting native metabolism toward ethanol production, as well as heterologous expression of ethanol production pathways in *C. thermocellum*; the improvements to ethanol yield and titer of these various approaches have been previously summarized [4].

Recently, four proteins from a strain of *Thermoanaerobacterium saccharolyticum* engineered for high levels of ethanol production (strain M1442, Herring et al. [5])—namely, an NADPH-dependent alcohol dehydrogenase (AdhA), the NADH-dependent reduced ferredoxin:NADP+ oxidoreductase complex (NfnAB), and a mutant bifunctional alcohol dehydrogenase (AdhE^G544D^)—were introduced into wild-type *C. thermocellum*, to improve ethanol yield, titer, and production rate [4]. However, the maximum ethanol titer achieved by this engineered *C. thermocellum* strain (LL1319) was only 15 g/L, which is far short of the 70 g/L ethanol titer that engineered *T. saccharolyticum* (strain M1442) is capable of producing [5].

In both *C. thermocellum* and *T. saccharolyticum*, the oxidative decarboxylation of pyruvate to acetyl-CoA is primarily catalyzed by a pyruvate ferredoxin oxidoreductase (PFOR) enzyme or enzyme complex [6–9]. In *C. thermocellum*, there are five candidates (Table 1) [10]; of these, the *pfor*s encoded by the genes *Clo1313_0020*-*0023* and *Clo1313_1353*-*1356* were reported to be cumulatively responsible for approximately 80% of the PFOR activity [10]. However, it is not known which of these five *pfor*s is important for ethanol production. In addition, prior to this work, all strains of *C. thermocellum* that had been engineered to produce ethanol with the *pfor* pathway have relied on the native *pfor*s; among these strains, the maximum ethanol titers observed were around 25 g/L, suggesting therefore that the native *pfor*s may not be capable of supporting ethanol production beyond that titer. By comparison in *T. saccharolyticum*, there are six genes annotated as putative *pfor*s, and deletion studies have shown that the *pforA* gene (*Tsac_0046* in strain DSM 8691) encodes the primary Pfor protein in *T. saccharolyticum*, as evidenced by the significant decrease in PFOR activity and ethanol production in strains which had the *pforA* gene deleted [11]. These results suggested that the *pforA*, unlike the *C. thermocellum pfor*s, was capable of supporting ethanol production to high titers (> 40 g/L).

**Table 1:** Gene names and locus numbers for five annotated *C. thermocellum pfor* genes or gene clusters

*pfor* name	DSM 1313 locus identifiers
*pfor1*	*Clo1313_0020*-*0023*
*pfor2*	*Clo1313_0382*-*0385*
*pfor3*	*Clo1313_0673*
*pfor4*	*Clo1313_1353*-*1356*
*pfor5*	*Clo1313_1615*-*1616*

Another important component of the pyruvate to ethanol pathway that utilizes a Pfor enzyme is ferredoxin, which is responsible for electron transfer from pyruvate to nicotinamide cofactors, in the context of ethanol production [12]. Previous work suggests that *C. thermocellum* ferredoxins are compatible with *T. saccharolyticum* ferredoxin-utilizing enzymes [13]. However, given that they play an important role in electron transfer, and that it is possible that some ferredoxins work better than others with certain enzymes, we decided to include the introduction of *T. saccharolyticum* ferredoxin into *C. thermocellum* in this study. We chose the Tsac_2084 ferredoxin because of its location adjacent to *adhA* and *nfnAB* (*Tsac_2087* and *Tsac_2086*-*2085*, respectively) on the chromosome; given that these latter two enzymes were previously shown to be important for ethanol production, both in *T. saccharolyticum* [14, 15] and *C. thermocellum* [4], this ferredoxin seemed to be a reasonable choice.

**Fig. 1:**
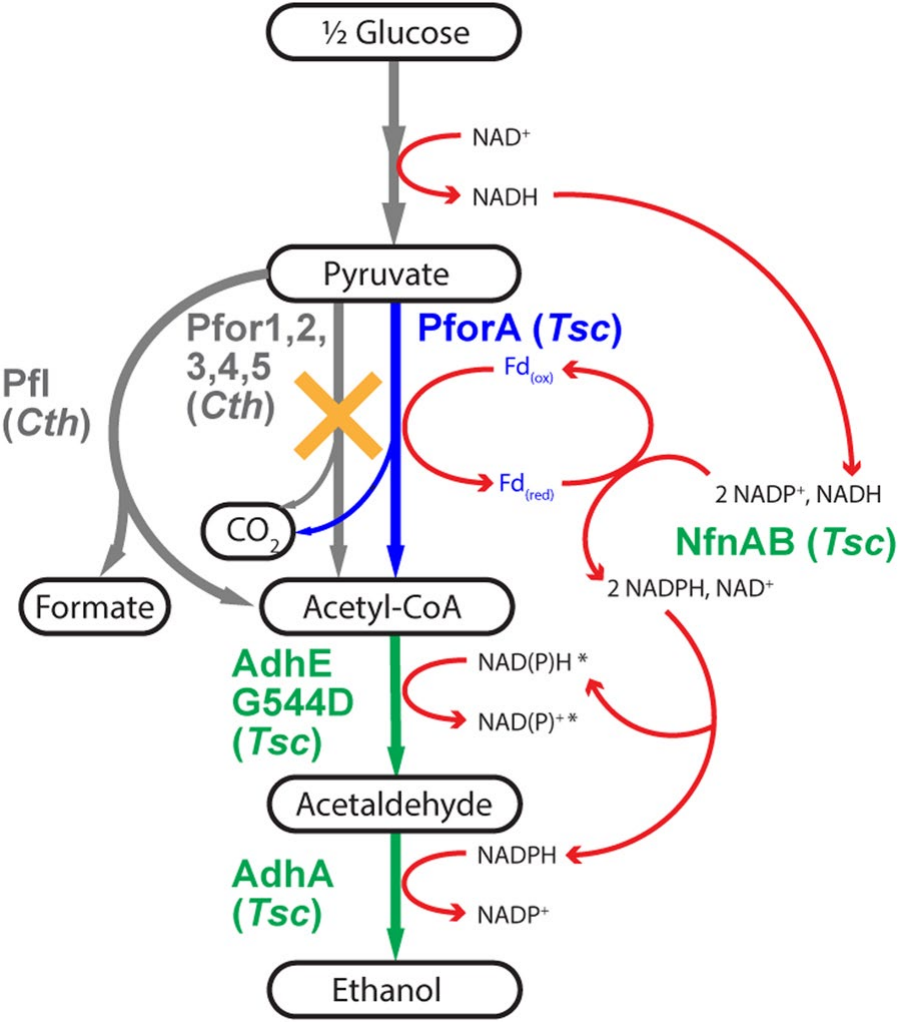
Pyruvate to ethanol production in *C. thermocellum* using the *T. saccharolyticum* pyruvate to ethanol pathway; this figure is adapted from Hon et al. [4]. Metabolites and products are colored black. Native genes (*Cth*) and the pathways they correspondingly catalyze are colored gray; *T. saccharolyticum* genes (*Tsc*) that were previously introduced in Hon et al. [4], as well as the reactions that they catalyze, are colored green. Red arrows represent electron flux. The *T. saccharolyticum pforA* and ferredoxin introduced in this study are depicted in blue. The orange cross represents the pathways that were deleted in this study

In this study, we hypothesized that introducing the *T. saccharolyticum pfor*A would improve ethanol production in a strain of *C. thermocellum* that had been previously engineered with other *T. saccharolyticum* ethanol production enzymes (i.e., strain LL1319). We also investigated whether the introduced PforA protein would be able to sustain ethanol production after the five known *C. thermocellum* Pfor enzyme and enzyme complexes were deleted (Fig 1). We also sought to determine the importance of *T. saccharolyticum* ferredoxin for ethanol production. We tested this by integrating *T. saccharolyticum pforA* (with or without the ferredoxin *Tsac_2084*) into *C. thermocellum* strains, and evaluating the effects that these genes had on enzyme activity, and on ethanol yield, titer, and maximum ethanol production rate, on soluble carbon sources as well as high loadings of crystalline cellulose.

## Methods

### Strain and plasmid construction

Table 2 lists all strains used and constructed in this study, and the plasmids used for integrating and deleting genes of interests. All plasmids were constructed via isothermal assembly [16] using a commercial kit sold by New England Biolabs (NEBuilder^®^ HiFi DNA Assembly Master Mix, catalog number E2621). Purification of plasmid DNA or PCR products for cloning was done using commercially available kits from Qiagen, Zymo Research, or New England Biolabs. *C. thermocellum* strains were transformed using previously described methods [17]. Plasmid DNA destined for transformation into *C. thermocellum* was purified from *Escherichia coli* BL21 derivative strains (New England Biolabs catalog number C2566) to ensure that the DNA was properly methylated [18].

**Table 2:** List of strains and integration/deletion plasmids used in this study

Strains/plasmids	Organism	Description	Accession number	References or source
*E. coli* T7 express	*Escherichia coli*	*fhuA2 lacZ::T7 gene1 [lon] ompT gal sulA11 R(mcr*-*73::miniTn10*–*TetS)2 [dcm] R(zgb*-*210::Tn10*–*TetS) endA1 Δ(mcrC*-*mrr)114::IS10*		New England Biolabs (Ipswich, MA)
M1442	*T. saccharolyticum*	Engineered and evolved *T. saccharolyticum*	SRA233073	[5]
LL1004	*C. thermocellum*	DSM 1313	CP002416	DSMZ
AG929	*C. thermocellum*	DSM1313 ∆*hpt* ∆*clo1313_0478*	SRP097241	[4]
LL1319	*C. thermocellum*	AG929 P_Clo1313_2638_::*adhA*(*Tsc*)-*nfnAB*(*Tsc*)-*adhE*^G544D^ (*Tsc*)	SRP101300	[4]
LL1565	*C. thermocellum*	AG929 *Clo1313_2637::P*_*pforA*(*Tsc*)_*pforA*(*Tsc*) -*fd*(*Tsc*)	SRP144031	This study
LL1391	*C. thermocellum*	LL1319 *Clo1313_2637::P*_*pforA*(*Tsc*)_*pforA*(*Tsc*)-*fd*(*Tsc*)	SRP141156	This study
LL1566	*C. thermocellum*	LL1319 *Clo1313_2637::P*_*pforA*(*Tsc*)_*pforA*(*Tsc*)	SRP144035	This study
LL1436	*C. thermocellum*	LL1391 Δ*Clo1313_0020*-*0023*	SRP144013	This study
LL1437	*C. thermocellum*	LL1391 Δ*Clo1313_1353*-*1356*	SRP144038	This study
LL1438	*C. thermocellum*	LL1436 Δ*Clo1313_1353*-*1356*	SRP144037	This study
LL1567	*C. thermocellum*	LL1437 Δ*Clo1313_0020*-*0023*	SRP144045	This study
LL1568	*C. thermocellum*	LL1438 Δ*Clo1313_0673*	SRP144054	This study
LL1569	*C. thermocellum*	LL1568 Δ*Clo1313_0382*-*0385*	SRP144051	This study
LL1570	*C. thermocellum*	LL1569 Δ*Clo1313_1615*-*1616*	SRP144049	This study
LL1556	*C. thermocellum*	DSM1313 Δ*hpt* Δ*Clo1313_0020*-*0023 P*_*rpi*_*: kivd*_*LL*_ – *P*_*pck*_*: ilvBNC*_*CT*_ – *P*_*ilvD*_*: ilvD*_*CT*_	SRP144036	This study
LL1559	*C. thermocellum*	DSM1313 Δ*hpt* Δ*Clo1313_0382*-*0385*	SRP144040	This study
LL1563	*C. thermocellum*	DSM1313 Δ*hpt* Δ*Clo1313_0673*		This study
LL1564	*C. thermocellum*	DSM1313 Δ*hpt* Δ*Clo1313_1353*-*1356*		This study
LL1560	*C. thermocellum*	DSM1313 Δ*hpt* Δ*Clo1313_1615*-*1616*	SRP144039	This study
pJGW37		*C. thermocellum* expression plasmid		[19]
pSH105		*P*_*enolase*_*pforA*(*Tsc*)-*fd*(*Tsc*) integration vector	MH245114	This study
pSH106		*P*_*pforA*(*Tsc*)_*pforA*(*Tsc*)-*fd*(*Tsc*) integration vector	MH245115	This study
pSH107		*P*_*Athe_2105*_*pforA*(*Tsc*)-*fd*(*Tsc*) integration vector	MH245116	This study
pSH121		*P*_*pforA*(*Tsc*)_*pforA*(*Tsc*) integration vector	MH245113	This study
pDGO77		Clo1313_0020-0023 deletion vector	MH245117	This study
pDGO78		Clo1313_0673 deletion vector	MH245118	This study
pSH116		Clo1313_1353-1356 deletion vector	MH245112	This study
pSH130		Clo1313_0382-0385 deletion vector	MH245110	This study
pSH131		Clo1313_1615-1616 deletion vector	MH245111	This study

### Media preparation and culture conditions

All reagents used in this study were of molecular grade and obtained from either Sigma Aldrich or Fisher Scientific, unless otherwise noted. *C. thermocellum* strains were grown at 55 °C under anaerobic conditions, either in conical tubes in an anaerobic chamber (Coy Laboratory Products, Grass Lakes, MI), with previously described environmental conditions [4], or in sealed 150 mL serum bottles that were prepared and inoculated as previously described [4].

Complex medium (CTFUD) was prepared as previously described and used to culture *C. thermocellum* cells in preparation for transformations, or for harvesting genomic DNA for strain resequencing.

The defined medium (MTC-5) was prepared as previously described [4] and used for all other purposes. Cellobiose was used as the main carbon source, unless otherwise noted. For MTC-5, 5 g/L cellobiose was used for routine culture and growing cells for gene expression analyses (see "Measuring gene expression") or enzyme assays (see "Enzyme assays"). Cellobiose concentrations of 20 g/L and 50 g/L were used for fermentation end product analyses; when 50 g/L cellobiose was used, the concentrations of pyridoxamine dihydrochloride, P-aminobenzoic acid, D-biotin, and vitamin B12 used were doubled to a final concentration of 0.04 g/L, 0.008 g/L, 0.008 g/L, and 0.004 g/L, respectively.

Specific growth rates, measured on MTC-5 medium with 5 g/L cellobiose as the main carbon source and using a microplate reader, were determined as previously described [20].

### Measuring gene expression

Gene expression was determined using reverse transcription quantitative PCR (RT-qPCR); Additional file 1: Table S1 lists the primers used for RT-qPCR. Cultures were grown on MTC-5 medium to mid-exponential phase (OD_600_ between 0.6 and 1.0); cultures were then processed as previously described [4]. Gene expression was normalized against *C. thermocellum recA* expression [21] to allow comparison across strains, as was previously done [4, 22, 23].

Translation initiation efficiencies were calculated using an online calculator from the Howard M. Salis group website (URL: https://salis.psu.edu/software/reverse) [24, 25].

### Enzyme assays

Cell cultures were grown, harvested, stored, and lysed to obtain cell-free extract as previously described [4]. Protein concentrations in the cell-free extracts were determined using Bio-Rad (Hercules, CA) protein dye assay reagent, with bovine serum albumin used as a protein standard.

All enzyme assays were performed at 55 °C and at pH 7.0 under anaerobic conditions in an anaerobic chamber (Coy Laboratory Products, Grass Lakes, MI).

Pyruvate ferredoxin oxidoreductase (PFOR) activity was assayed by the reduction of benzyl viologen instead of methyl viologen, a modification of a previously described protocol [11]. The reduction of benzyl viologen was monitored at a wavelength of 578 nm, and an extinction coefficient of 7.8 mM^−1^ cm^−1^ was used to calculate activity [26]. The assay mixture contained 100 mM Tris–HCl, 5 µM FeSO_4_, 0.5 mM DTT, 2 mM MgCl_2_, 0.4 mM coenzyme A, 0.4 mM thiamine pyrophosphate, and 1 mM benzyl viologen dichloride. Cell extract was added to this assay mixture first to establish a baseline; the reaction was then started by adding 2 mM sodium pyruvate. The final volume for all biochemical assays was 1200 µL.

### High solids fermentations

Bioreactor experiments were carried out as previously described [27], with an initial working volume of 1 L. MTC-5 medium [4] was modified to have 5 g/L initial urea instead of 2 g/L as previously reported, Vitamin and trace mineral concentrations were increased to 4× and 5× of previously reported values [4]. 100 g/L crystalline cellulose (Avicel PH105) was used as the main carbon source. Bioreactors were inoculated with 5 mL of an overnight culture (0.5% v/v inoculum) that was grown on MTC-5 medium modified to contain 5 g/L MOPS sodium salt and to use 5 g/L Avicel as the main carbon source. pH was maintained at 7.00 ± 0.05 by the automatic addition of 4 N KOH.

### Analytical methods

The fermentation products were measured by high-pressure liquid chromatography (HPLC) as previously described [27]. For tube and serum bottle cultures, the results were normalized against an internal standard (MOPS buffer) to account for variation due to sample processing and handling. Headspace gas composition for serum bottle fermentations was measured as previously described [23].

To quantify extracellular amino acids, samples were first derivatized with a commercially available derivatization reagent (Accq-Tag Chemistry kit, catalog number WAT052875, Waters Corporation, Milford, MA). The derivatized samples were run on an HPLC equipped with the Waters AccQ∙Tag column (part number WAT052885, Waters Corporation, Milford, MA) with fluorescence detection, using an excitation wavelength of 250 nm and emission wavelength of 395 nm, following the manufacturer’s recommended instrument method. Sample preparation was performed according to the manufacturer’s instructions (manual number WAT052874, Rev 1, Waters Corporation, Milford MA).

Residual cellulose (Avicel PH105) concentration was determined via quantitative saccharification [28]. Pellet nitrogen (a proxy for cell biomass) was measured with a Shimadzu TOC-V CPH elemental analyzer with TNM-1 and ASI-V modules (Shimadzu Corp., Columbia, MD), as previously described [28, 29].

Volumetric ethanol production from bioreactor experiments was determined by fitting ethanol production data points for each fermentation with the five-parameter sigmoidal Richards equation [30], and calculating the first derivative of each fermentation’s fitted Richards equation.

### Sequencing and resequencing

Routine Sanger sequencing of plasmids was performed by Genewiz. Inc., with at least twofold coverage of the cloned regions. Whole genome resequencing of strains was performed by the Department of Energy Joint Genome Institute, using the Illumina Miseq sequencing platform, with an average of 100-fold coverage. Sequencing data were analyzed with the CLC Genomics workbench, using strain LL1319 as the reference genome (accession number SRP101300). Accession numbers for strains and plasmids are listed in Table 2. A summary of the resequencing results can be found in Additional file 2.

## Results

### Choosing a promoter to drive *pforA* expression

*Thermoanaerobacterium saccharolyticum pforA* and ferredoxin were integrated into the *C. thermocellum* genome in strain LL1319, at a location immediately downstream of *Clo1313_2637*, and before the putative promoter for the *Clo1313_2635* gene (Additional file 1: Figure S1). This was done so as to locate the new operon close to the previously introduced *T. saccharolyticum adhA*-*nfnAB*-*adhE*^*G544D*^ operon [4], without disrupting a putative peroxiredoxin two-gene cluster (*Clo1313_2638*-*2637*). Three promoters were tested to drive expression of the *pforA*–ferredoxin operon: the previously described *C. thermocellum* enolase promoter [31], the *T. saccharolyticum pforA* promoter, and the *Athe_2105* promoter from *Caldicellulosiruptor bescii* [19] with a modified ribosome binding site [24]. Promoter sequences and predicted translation initiation efficiencies of *pforA* are reported in Additional file 1: Table S2.

**Fig. 2:**
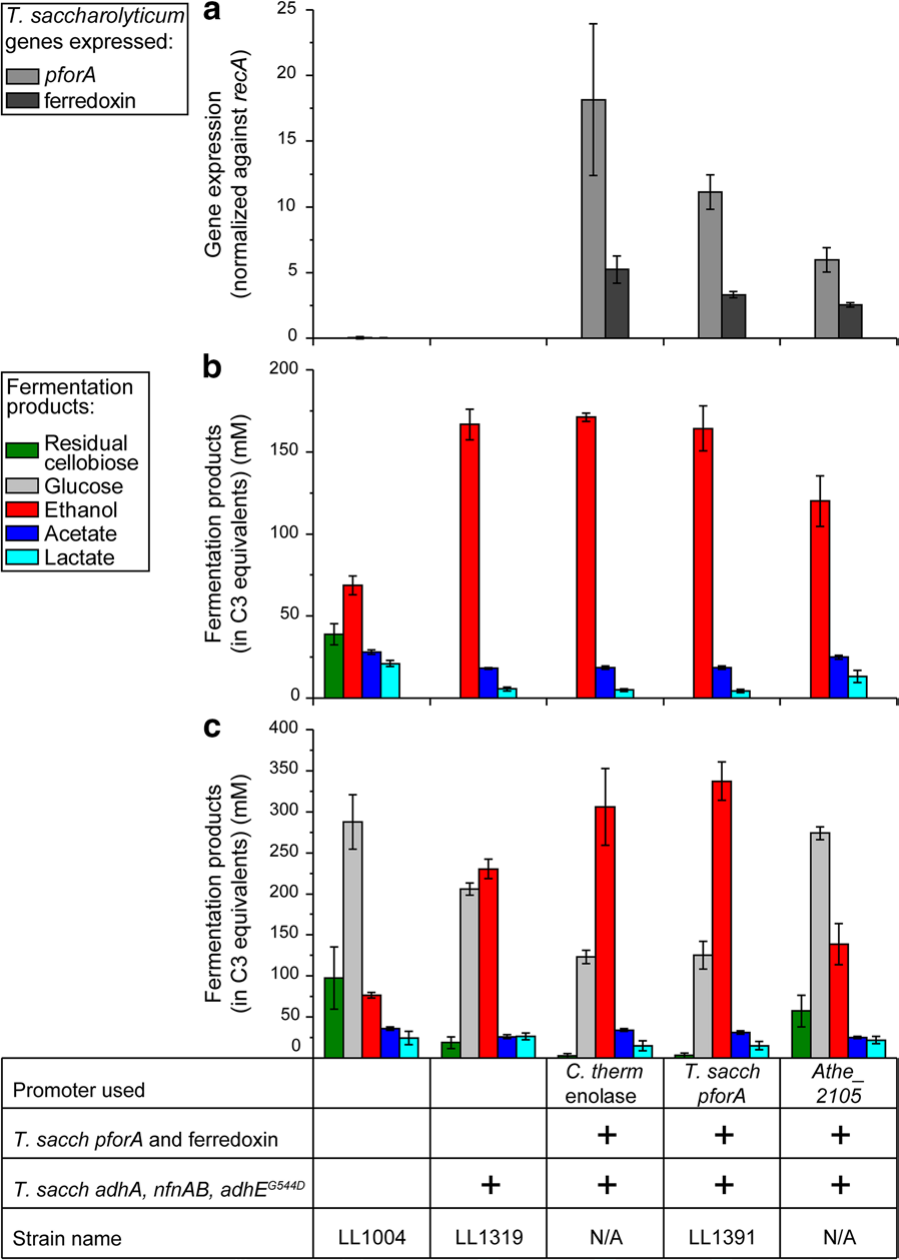
**a** Relative expression levels (normalized against *C. thermocellum recA* expression) of *T. saccharolyticum pforA* and ferredoxin. **b**, **c** Fermentation products of five *C. thermocellum* strains. Cultures were grown in 15 mL tubes with 5 mL of MTC-5 medium, with 60 ± 2 mM (~ 20 g/L) (**b**) or 151 ± 3 mM (~ 52 g/L) (**c**) initial cellobiose, for 72 h and 168 h, respectively, at 55 °C. Error bars represent one standard deviation (*n* = 3 for **a** and **b**, *n* = 5 for **c**)

*T. saccharolyticum pforA* and ferredoxin expression was observed in all three operon configurations (Fig. 2a), but not in wild-type *C. thermocellum* and the parent strain, LL1319, as expected. In general, we observed that ferredoxin expression was lower than that of the *pforA* gene. It was also observed that the *C. thermocellum* enolase promoter resulted in the highest level of gene expression, followed by the *T. saccharolyticum pforA* promoter, with the modified *Athe_2105* promoter giving the lowest level of expression. However, we also observed that expression was more variable with the *C. thermocellum* enolase promoter than with the two heterologous promoters. The variation observed with the native enolase promoter is similar to what we have previously observed for *lacZ* expression [31].

**Fig. 3:**
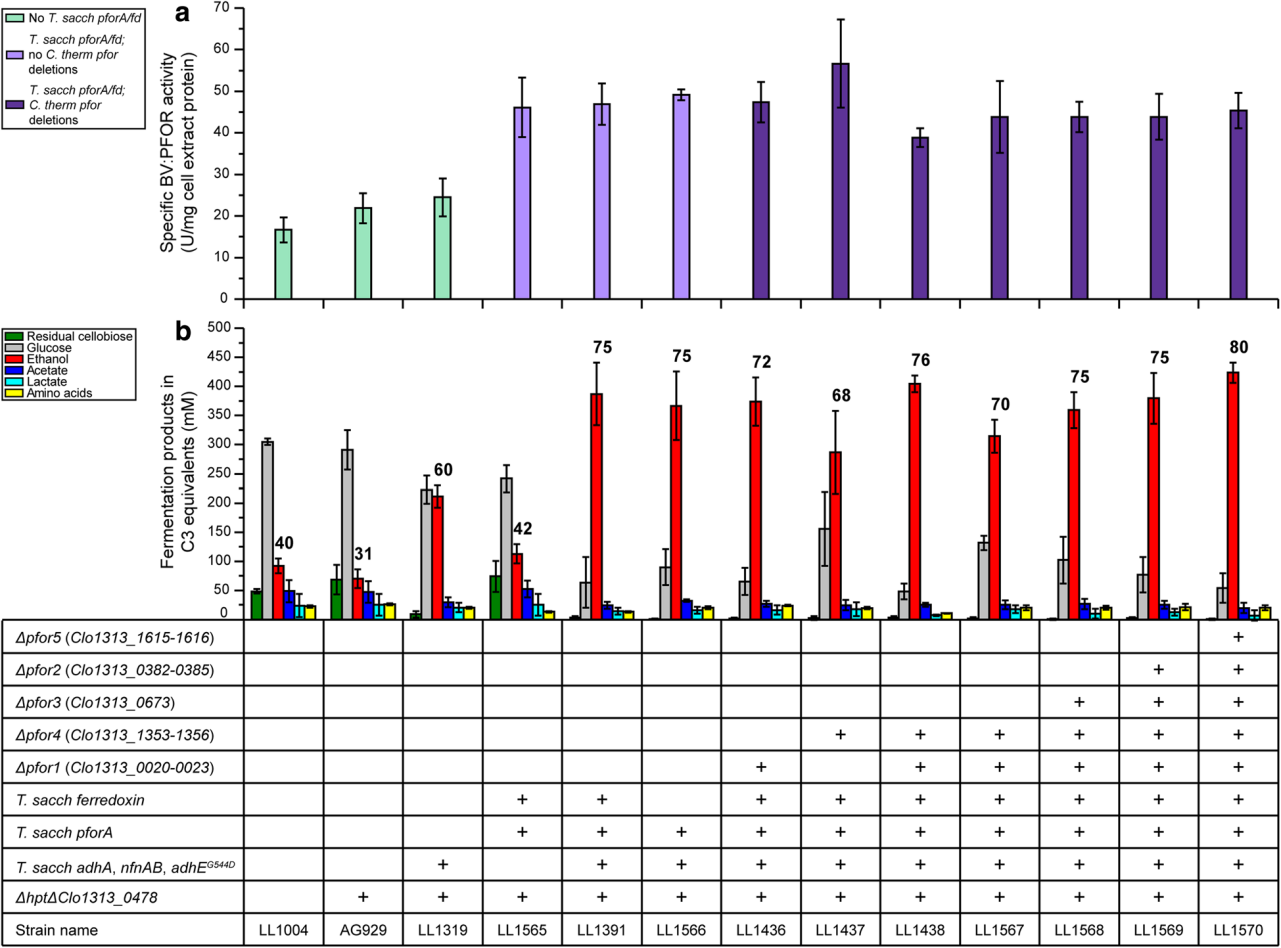
Specific BV:PFOR activity (**a**) and fermentation products (**b**) for 13 strains of *C. thermocellum*. The plus sign indicates the presence of a genetic modification in a strain. For enzyme activity, 1 unit (U) is equivalent to the formation of 1 µmol of product per minute. For the fermentations, cultures were grown in sealed serum bottles with 20 mL of MTC-5 medium with 145 ± 2 mM initial cellobiose (~ 50 g/L) for 168 h at 55 °C, with 180 rpm shaking. Values over the ethanol (red) columns represent the metabolic yield of ethanol as a percentage of theoretical maximum (assumes that a maximum of 2 moles of ethanol can be produced from 1 mole of glucose or glucose equivalent). Error bars represent one standard deviation (*n* ≥ 2 for enzyme specific activity, *n* ≥ 3 for fermentation products). Raw data and growth rates are presented in Additional file 1: Table S3; individual quantification of extracellular amino acid production is shown in Additional file 1: Table S4

Fermentation product profiles for the *C. thermocellum* strains using the native enolase and *T. saccharolyticum pforA* promoters were indistinguishable from the parent strain LL1319 on 20 g/L initial cellobiose. The strain that used the *C. bescii Athe_2105* promoter, however, showed an unexpected decrease in ethanol production (Fig. 2b). On 52 g/L initial cellobiose, we again observed that the *Athe_2105* promoter-containing strain exhibited reduced ethanol production compared to the parent strain LL1319 (Fig. 2c), unlike the other two strains that contained the *T. saccharolyticum pforA* and ferredoxin; this strain was thus excluded from further investigations. Between the two other strains, both showed comparable improvements to ethanol production over the parent strain LL1319, with the *T. saccharolyticum pforA* promoter-driven strain showing slightly higher ethanol titers. Given that the *T. saccharolyticum pforA* promoter resulted in the highest levels of ethanol production of the three promoters tested, and that its use avoids duplicating native DNA sequences (which can lead to unintended recombination events and complicate the analysis of resequencing data), we proceeded forward with this strain and designated it as strain LL1391 (Table 2). Subsequent fermentations confirmed that strain LL1391 produced more ethanol than strain LL1319 (Fig. 3b), which may be attributed to a significant increase in the BV:PFOR specific enzyme activity in strain LL1391 (unpaired two-tailed *t* test, p = 0.0045) (Fig. 3a).

### The effects of *T. saccharolyticum pforA* and ferredoxin expression on ethanol production

Strain LL1319 contains four *T. saccharolyticum* genes from previous strain development [4]; we therefore investigated whether the improvements in ethanol production were dependent on the presence of the previously introduced *T. saccharolyticum adhA, nfnAB, and adhE*^*G544D*^ genes. Strain LL1565 was created by integrating *T. saccharolyticum pforA* and ferredoxin, driven by the *T. saccharolyticum pforA* promoter (plasmid pSH106, Table 2) into strain AG929 (the parent strain of strain LL1319). A significant increase in BV:PFOR specific activity in strain LL1565 was observed, relative to strain AG929 (unpaired two-tailed t test, p = 0.0064); the measured specific activity of strain LL1565 was comparable to activity levels measured in strain LL1391 (Fig. 3a), indicating that the introduced *T. saccharolyticum* PforA protein was present and active in the strain. Ethanol production with strain LL1565 appeared to be slightly improved relative to parent strain AG929, although the metabolic yield and final titers were significantly lower than that of strain LL1319 and by extension that of LL1391 (Fig. 3b), suggesting that the improvement in ethanol production from introducing *pforA* is dependent on the presence of the other *T. saccharolyticum* ethanol production pathway genes.

To determine whether the *T. saccharolyticum* ferredoxin was necessary for the improvements in ethanol production, *T. saccharolyticum pforA* alone was integrated into strain LL1319 at the same locus as was done in strain LL1391 to create strain LL1566 (using plasmid pSH121, see Table 2).  The BV:PFOR specific enzyme activity for strain LL1566 was no different from that observed in LL1391, as expected; fermentation products for the two strains were also similar, suggesting that the introduced *T. saccharolyticum* PforA protein was responsible for the improvements in ethanol production (Fig. 3). We attempted to introduce the *T. saccharolyticum* ferredoxin on its own into strain LL1319, but were not successful. Given that there appeared to be no detrimental effects in ethanol production due to the presence of *T. saccharolyticum* ferredoxin (Fig. 3), and that the ferredoxin is important in the production of ethanol as an electron carrier, we decided to retain it in subsequent strains (see Table 2 for strain lineage).

### The effect of deleting native *pfor*s on ethanol production

The introduction and expression of *T. saccharolyticum pforA* have thus far been associated with an increase in BV:PFOR specific activities, and an increase in ethanol titer and metabolic yield (Fig. 3). However, the strains evaluated so far still contain the five native Pfor-encoding genes and gene clusters (Table 1). To better determine whether the improvements in ethanol production were due to the introduced *T. saccharolyticum* PforA protein, we deleted all five *C. thermocellum pfor* gene clusters in an iterative manner.

Previous work suggested that *pfor1* (*Clo1313_0020*-*0023*) and *pfor4* (*Clo1313_1353*-*1356*) encoded for the main Pfor protein complexes in *C. thermocellum* [10]. Further support for *pfor1* and *pfor4* encoding for important Pfor complexes in *C. thermocellum* was found when it was observed that BV:PFOR specific activity decreased by ~ 80% relative to wild-type *C. thermocellum* (Additional file 1: Figure S2) when either *pfor1* or *pfor4* was deleted in *C. thermocellum* (strain LL1556 and LL1564). Strains containing a deletion of either *pfor2* or *pfor5* did not show any significant differences in BV:PFOR specific activity, suggesting that they were not important for PFOR activity in *C. thermocellum*, or that pyruvate was not the primary substrate for these enzymes. The deletion of *pfor3*, which bears the most similarity to the *T. saccharolyticum pforA*, also resulted in ~ 40% decrease in specific BV:PFOR activity. Given these observations, *pfor1 and pfor4* were therefore the first targets for gene deletion in strain LL1391.

Starting with strain LL1391 (wt strain expressing *T. saccharolyticum* pathway, including *pforA*), deletion of *pfor1* (strain LL1436) did not result in any significant effects on ethanol production or enzyme specific activity. Deletion of *pfor4* (strain LL1437), however, showed a decrease in ethanol yield and a large decrease in titer (Fig. 3b), despite very little change in BV:PFOR activity (LL1391 vs. LL1437, unpaired two-tailed *t* test, p = 0.23) (Fig. 3a).

Resequencing analyses subsequently revealed that LL1437 contained a 1207G > T mutation in the coding sequence for the *Clo1313_1483* gene that resulted in a G403* nonsense mutation in the amino acid sequence; excluding the targeted *pfor* deletions, there were no other differences between the genomes of the two strains. *Clo1313_1483* is annotated as encoding a predicted pyrroloquinoline quinone-associated protein, and previous work suggests that it is expressed in *C. thermocellum* strain ATCC27405 [32, 33]; however, its function in *C. thermocellum* strain DSM1313 is unknown.

To compare the effect of the *Clo1313_1483* mutation vs. *pfor4* deletion, we constructed two *pfor1*/*pfor4* double deletion strains: LL1438 (LL1436 with *pfor4* deleted) and LL1567 (LL1437 with *pfor1* deleted). Since neither of these strains showed any significant difference in ethanol production relative to their respective parent strains (LL1438 vs. LL1436 and LL1567 vs. LL1437), the difference in ethanol production between LL1436 and LL1437 is likely due to the *Clo1313_1483* mutation, and not the effect of the *pfor4* deletion.

**Fig. 4:**
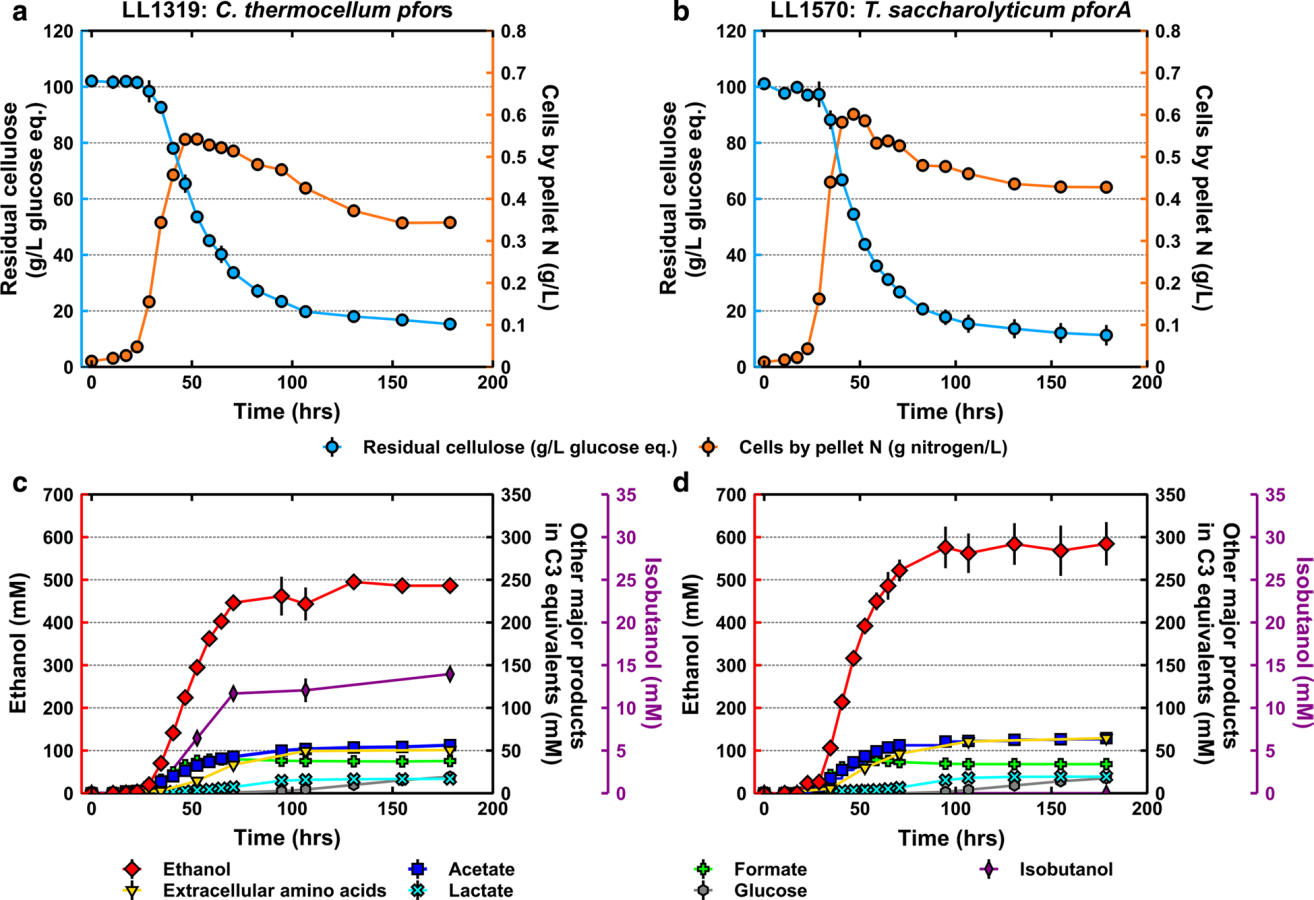
Comparison of the effect of fermentation with native *C. thermocellum pfors* (strain LL1319) or *T. saccharolyticum pforA* (strain LL1570). Substrate utilization as residual cellulose (light blue) and cells by pellet nitrogen (orange) versus fermentation times for strains LL1319 (**a**) and LL1570 (**b**). Major fermentation products—ethanol (red), acetate (dark blue), lactate (light blue), formate (light green), glucose (gray), isobutanol (purple), and extracellular amino acids (yellow) from the same fermentation runs for strains LL1319 (**c**) and LL1570 (**d**). Error bars represent mean absolute deviation (*n* = 2 fermentations). The data shown is for representative fermentations. Data for additional replicates is presented in Additional file 1: Table S5 and Figure S3. See Additional file 3 for tabular presentation of the data

The double *pfor1*/*pfor4* deletion strain, LL1438, was still able to sustain the improved ethanol production observed in strain LL1391. To eliminate the possibility that this was due to the remaining three *C. thermocellum* Pfor enzymes compensating for the *pfor1* and *pfor4* deletions, the genes encoding for *pfor3*, *pfor2*, and *pfor5* (see Table 1 for gene numbers) were iteratively deleted to create strain LL1570. Strain LL1570 was able to produce 424 ± 13 mM (~ 20 g/L) of ethanol from 50 g/L of cellobiose, with a metabolic ethanol yield of 80% of the theoretical maximum (Fig. 4b; also see Additional file 1: Table S3), an improvement over the reference strain LL1319, which was previously reported to have achieved a maximum ethanol yield of 74% of theoretical maximum on 20 g/L cellobiose, and a maximum ethanol titer of 326 mM (~ 15 g/L) on 60 g/L Avicel (120). The maximum specific growth rate of strain LL1570 on cellobiose was unaffected relative to the starting strain, LL1319 (Additional file 1: Table S3), suggesting that deleting the native *pfor* genes did not result in any growth defects, and that the introduced *T. saccharolyticum pforA* complemented the deletions of these five native *C. thermocellum pfor* genes.

In strain LL1570, we would have expected that deletion of *pforA* would eliminate acetate and ethanol production and divert flux to lactate production (similar to what was observed for *pfor* deletions in *T. saccharolyticum* [11]). Despite several attempts to delete *pforA* in this strain, we were not successful. Although not conclusive, this negative result suggests that PFOR activity is essential in this strain, and that *pforA* is the source of that activity.

### Fermentation of high cellulose concentrations

To determine if replacing of the native *pfor*s with the *T. saccharolyticum pforA* and ferredoxin had improved the maximum ethanol titer and maximum volumetric production rate of ethanol, and to evaluate the performance of the strain on a cellulosic substrate, strains LL1319 and LL1570 were grown on 100 g/L Avicel microcrystalline cellulose.

Strains LL1319 and LL1570 consumed about the same amount of Avicel (Figs. 3b and 4a, see also Additional file 1: Table S5), but produced different amounts of ethanol. Strain LL1319 produced ethanol to a titer of 486 ± 5 mM (~ 22 ± 0.2 g/L) (Fig. 4c), for a metabolic yield of 45% of the theoretical maximum; the ethanol titer observed here was higher than previously reported [4], although it should be noted that the media composition was different. In contrast, strain LL1570 produced 551 ± 32 mM (25 ± 1.5 g/L) of ethanol (Fig. 4d, Additional file 1: Figure S3), for a metabolic yield of 54% of theoretical maximum. The results provide further evidence that *T. saccharolyticum pforA* improved ethanol yield and titer (unpaired two-tailed *t* test; p = 0.003 for ethanol yield, p = 0.02 for ethanol titer). The differences in volumetric productivity of ethanol between strains LL1319 and LL1570 (0.66 ± 0.03 g L^−1^ h^−1^ and 0.70 ± 0.12 g L^−1^ h^−1^, respectively) were not statistically significant (unpaired two-tailed *t* test; p = 0.338) (Additional file 1: Table S5). The ethanol titer of 25 g/L for strain LL1570 was very similar to that produced by another engineered strain of *C. thermocellum*, LL1210 (Δ*hpt* Δ*hydG* Δ*ldh* Δ*pfl* Δ*pta*-*ack adhE*(D494G)), which was generated by eliminating the native competing carbon and electron pathways, followed by strain adaptation over ~ 2500 generations to increase growth rate, and which was reported to produce 27 g/L of ethanol from 95 g/L of Avicel [34]. The byproduct concentrations (organic acids and total extracellular amino acids) in both strains LL1319 and LL1570 were similar, except for isobutanol production, which decreased to below our limit of quantification (0.1 mM) in strain LL1570 (Fig. 4d) (strain LL1319 produced a maximum isobutanol titer of ~ 14 mM; see Fig. 4c); this suggests that one of the deleted *C. thermocellum pfor*s may be involved in the biosynthesis of isobutanol. Fermentation results from a set of *C. thermocellum* strains that contain a deletion of one of the five annotated *pfor*s suggest that it is *pfor4* that is associated with isobutanol production (Additional file 1: Figure S4).

### Discussion and conclusion

In this work, we investigated the effects of *T. saccharolyticum pforA* and ferredoxin on ethanol production in *C. thermocellum*. There was no effect from expressing *T. saccharolyticum* ferredoxin in *C. thermocellum*. It is known that ferredoxins from one organism can often transfer electrons to proteins from another organism [35], so it would not be surprising if one of the native *C. thermocellum* ferredoxins was sufficient for electron transfer from *T. saccharolyticum* Pfor protein.

Introducing just the *T. saccharolyticum pforA* did not improve ethanol titer, but it slightly shifted the ethanol to acetate ratio in favor of ethanol production. When the *T. saccharolyticum pforA* was expressed alongside the previously introduced *T. saccharolyticum adhA*, *nfnAB*, and *adhE*^*G544D*^ genes, ethanol production improved. These observations support the hypothesis that *pforA* is an important component of the *T. saccharolyticum* pyruvate-to-ethanol pathway. Furthermore, the *pforA* from *T. saccharolyticum* was able to functionally complement the deletion of the five annotated *C. thermocellum pfor* genes.

Isobutanol production in strain LL1570 was reduced below our limit of quantification (0.1 mM). Fermentation data from a set of *C. thermocellum* strains with single deletions of each of the five annotated *pfor*s points to *pfor4* being responsible for isobutanol production. Given the interest in producing isobutanol either for use as a biofuel or as a feedstock chemical [36]; the knowledge that *pfor4* is necessary for isobutanol production could be beneficial to further improve its production from cellulosic substrates.

The ability to use a *T. saccharolyticum* promoter to drive the expression of the *pforA*-ferredoxin operon is relevant to future work involving gene expression in *C. thermocellum*. Whereas native *C. thermocellum* promoters have been characterized [31], with the enolase promoter being used successfully in this study to express *T. saccharolyticum pforA*, these promoters may still be subject to native transcriptional regulation, and therefore may not be suitable if constitutive gene expression is desired. It should be noted that other examples of heterologous promoters being used in *C. thermocellum* have been reported [19]. As heterologous gene expression becomes more prevalent in *C. thermocellum* [4, 22, 37], it will become increasingly necessary to develop libraries of non-native or synthetic genetic tools to avoid excessive duplicating of native DNA elements, which could contribute to genome instability.

Having now introduced six genes from the *T. saccharolyticum* pyruvate to ethanol pathway into *C. thermocellum*, we observe that there is still a ~ 40 g/L difference between the maximum ethanol titers achieved by engineered *C. thermocellum* (25–30 g/L) [34] and those achieved by engineered *T. saccharolyticum* (60–70 g/L) [5], suggesting that there remains more work to be done. With regard to the Pfor-catalyzed conversion of pyruvate to acetyl-CoA, one consideration is that the Pfor enzyme functions in tandem not only with ferredoxin, but also with ferredoxin:NAD(P)^+^ oxidoreductase (Fnor). It is possible that titer limitations in engineered *C. thermocellum* are due to an un-optimized Pfor–ferredoxin–Fnor module. Some promising directions for further research include characterizing the Pfor–ferredoxin–Fnor modules of *C. thermocellum* and *T. saccharolyticum* in more detail, or engineering the module with better enzymes or through protein engineering of the existing enzymes to overcome possible substrate or cofactor inhibitions [38, 39].

## Additional files

**Additional file 1.** Supplementary material for this study, including primer sequences (**Table S1**), promoter sequences (**Table S2**), fermentation data (**Table S3**, **Table S4**, **Table S5**, **Figure S3** and **Figure S4**), chromosomal maps (**Figure S1**) and enzyme assay data (**Figure S2**).

**Additional file 2.** List of mutations observed among the strains described in this study

**Additional file 3.** Tabular presentation of the data from fermentation of high cellulose concentrations.
