# Supplementary material for: Expressing the Thermoanaerobacterium saccharolyticum pforA in engineered Clostridium thermocellum improves ethanol production
Source: Biotechnol Biofuels. 2018 Sep 6;11:242. doi: 10.1186/s13068-018-1245-2 (PMC6125887; doi:10.1186/s13068-018-1245-2)
Supplement: Supplementary file 1 — Additional file 1. Supplementary material for this study, including primer sequences (Table S1), promoter sequences (Table S2), fermentation data (Table S3, Table S4, Table S5, Figure S3 and Figure S4), chromosomal maps (Figure S1) and enzyme assay data (Figure S2). [file 13068_2018_1245_MOESM1_ESM.docx]

**Additional figures**

| **Primer name** | **Gene Target** | **Oligo sequence (5' to 3')** |
| --- | --- | --- |
| pfor_F | *T. saccharolyticum pforA* (*Tsac_0046*) | ATCAAGCTTGGAATGGGTTG |
| pfor-R | *T. saccharolyticum pfor A* (*Tsac_0046*) | GCTGTTGGAGCCTTTGAGTC |
| XSH0587 | *T. saccharolyticum* ferredoxin (*Tsac_2084*) | TTGCGCTGCAGAATGC |
| XSH0588 | *T. saccharolyticum* ferredoxin (*Tsac_2084*) | CTCCTGTTGGGCATACTGG |
| recA F | *C. thermocellum recA* | TTTACGGCCAGGGTATTTCA |
| recA R | *C. thermocellum recA* | GCCAATCTTCTGACCGTTGT |

**Table S1.** List of primers used for measuring gene expression via RT-qPCR

| **Promoter name** | **Originating organism** | **Sequence (5’ → 3’)** | **Predicted translation initiation efficiency of *pforA* (arbitrary units)** |
| --- | --- | --- | --- |
| Enolase | *C. thermocellum* | GGAAATATTAAAATGGAAATGTTGAAAAAATGTTTTAAGATGGGTCATTATGGATAAAATATACTATGGTTTTGCAATAAATGCTTTCTATTAATTGGACTTTGTGGTAATATGGTAGAAGGATGCAGTGTTAATTTTTTAACATATAAAAATAAGCTATATGAAGGGAGAATGGAGA | 19524 |
| *pforA* | *T. saccharolyticum* | ATCACATAATATGACCAGCTCATAATTTTTATGAATAAATTTTTGTCAAAAAAATATTTTTATATTTTAATTATCTGTGATATAATTATCGTGGAACTGTTATATAAAGTCTAAAGGTATATATATAAGCATTTTCAGAAAACTGTTATATAATCGCTTAACCGCTGTTGTATAACAGTACATATTTGTTTATGCTGTATTATAATAGTTTGCTTATATATCCTTAGATTTAAATATAAATTTAAAAAGAGAGGGGAAATACA | 25669 |
| *Athe_2105* | *C. bescii* | TTCAACAACCAGAGACACTTGGGAAATAACATATCCACAGAGTTATCCACATTATCCACAGCTTATTTTTAAACAGAATGGATATTTGCTTATTGTGAATTGTGGATAATTTTGTGGAAAAAATTTTTGTGTAAATAAGTCGAAATTGGTGGTATATATATAATATCAAAACAAAACTTTCTACATAGGAGGGATGGTC | 10492 |

**Table S2.** Sequences for the promoters used in the strains represented in figures 1 and 2. Translation initiation efficiencies were determined using a free calculator that is available on the Salis lab website (URL <https://salislab.net/software/reverse>). The predicted translation initiation efficiency for the *T. saccharolyticum* ferredoxin in the three *pforA-*ferredoxin operons is 104429.7 arbitrary units. Predicted translation initiation was calculated using the 100 bps sequence upstream and downstream of a start codon (i.e. the “A” in the starting “ATG” is the 100^th^ base pair).

| Strain | Fermentation products (mmol) | | | | | |
| --- | --- | --- | --- | --- | --- | --- |
|  | Residual cellobiose | Glucose | Ethanol | Acetate | Lactate | Formate |
| MTC | 2.89 (0.03) | 0.05 (0.00) | 0.02 (0.04) | 0.00 (0.00) | 0.00 (0.00) | 0.00 (0.00) |
| LL1004 | 0.24 (0.02) | 3.05 (0.06) | 1.84 (0.26) | 0.98 (0.39) | 0.48 (0.40) | 0.13 (0.02) |
| AG929 | 0.34 (0.13) | 2.91 (0.34) | 1.41 (0.32) | 0.95 (0.37) | 0.51 (0.37) | 0.13 (0.05) |
| LL1319 | 0.05 (0.03) | 2.23 (0.24) | 4.22 (0.38) | 0.59 (0.17) | 0.41 (0.16) | 0.18 (0.03) |
| LL1565 | 0.37 (0.13) | 2.42 (0.23) | 2.26 (0.33) | 1.05 (0.29) | 0.50 (0.37) | 0.08 (0.01) |
| LL1391 | 0.02 (0.01) | 0.64 (0.44) | 7.74 (1.08) | 0.49 (0.12) | 0.29 (0.12) | 0.11 (0.02) |
| LL1566 | 0.01 (0.01) | 0.90 (0.31) | 7.33 (1.18) | 0.65 (0.04) | 0.32 (0.12) | 0.06 (0.01) |
| LL1436 | 0.01 (0.01) | 0.65 (0.23) | 7.48 (0.83) | 0.54 (0.10) | 0.33 (0.16) | 0.07 (0.03) |
| LL1437 | 0.01 (0.02) | 1.56 (0.63) | 5.74 (1.42) | 0.50 (0.18) | 0.35 (0.24) | 0.10 (0.05) |
| LL1438 | 0.02 (0.01) | 0.48 (0.13) | 8.09 (0.28) | 0.51 (0.07) | 0.15 (0.03) | 0.07 (0.03) |
| LL1567 | 0.01 (0.01) | 1.32 (0.12) | 6.29 (0.56) | 0.51 (0.15) | 0.36 (0.14) | 0.10 (0.03) |
| LL1568 | 0.00 (0.01) | 1.02 (0.40) | 7.18 (0.62) | 0.53 (0.17) | 0.20 (0.17) | 0.10 (0.03) |
| LL1569 | 0.01 (0.01) | 0.77 (0.31) | 7.59 (0.87) | 0.51 (0.13) | 0.25 (0.12) | 0.07 (0.04) |
| LL1570 | 0.00 (0.01) | 0.54 (0.25) | 8.47 (0.35) | 0.39 (0.18) | 0.14 (0.18) | 0.08 (0.05) |

**Table S3.** Fermentation products (in mmols) and maximum specific growth rate µ_max_ for the strains represented in Figure 2. Values in parentheses represent one standard deviation (n ≥ 3 for fermentation products, n = 6 for growth rate).

| Strain | Fermentation products (mmol) | | | | | Maximum specific growth rate µ_max_ (/h) |
| --- | --- | --- | --- | --- | --- | --- |
|  | Pyruvate | Malate | Succinate | Total amino acids | hydrogen |  |
| MTC | 0.00 (0.00) | 0.00 (0.00) | 0.01 (0.01) | 0.00 (0.00) | 0.00 (0.00) | N/A |
| LL1004 | 0.00 (0.00) | 0.07 (0.03) | 0.00 (0.00) | 22.68 (2.23) | 1.94 (0.19) | 0.49 (0.01) |
| AG929 | 0.00 (0.00) | 0.08 (0.03) | 0.00 (0.01) | 26.15 (1.49) | 1.56 (0.87) | 0.53 (0.01) |
| LL1319 | 0.00 (0.00) | 0.09 (0.03) | 0.01 (0.01) | 20.59 (1.77) | 1.08 (0.14) | 0.48 (0.02) |
| LL1565 | 0.00 (0.00) | 0.08 (0.04) | 0.00 (0.00) | 13.29 (1.38) | 2.06 (0.22) | 0.49 (0.04) |
| LL1391 | 0.00 (0.00) | 0.07 (0.04) | 0.01 (0.01) | 13.52 (1.28) | 0.83 (0.03) | 0.52 (0.03) |
| LL1566 | 0.00 (0.00) | 0.10 (0.01) | 0.01 (0.00) | 20.08 (2.65) | 1.08 (0.06) | 0.49 (0.01) |
| LL1436 | 0.00 (0.00) | 0.07 (0.03) | 0.01 (0.01) | 24.11 (1.15) | 1.02 (0.10) | 0.54 (0.01) |
| LL1437 | 0.00 (0.00) | 0.09 (0.02) | 0.00 (0.01) | 19.68 (2.08) | 1.02 (0.28) | 0.47 (0.02) |
| LL1438 | 0.00 (0.00) | 0.08 (0.01) | 0.00 (0.00) | 10.96 (0.43) | 0.76 (0.16) | 0.47 (0.02) |
| LL1567 | 0.00 (0.00) | 0.11 (0.02) | 0.01 (0.01) | 20.48 (4.47) | 0.93 (0.08) | 0.41 (0.02) |
| LL1568 | 0.00 (0.00) | 0.07 (0.04) | 0.01 (0.01) | 20.24 (3.22) | 0.89 (0.08) | 0.43 (0.01) |
| LL1569 | 0.00 (0.00) | 0.08 (0.04) | 0.01 (0.01) | 21.35 (5.48) | 0.83 (0.10) | 0.53 (0.02) |
| LL1570 | 0.00 (0.00) | 0.06 (0.03) | 0.00 (0.00) | 20.11 (4.13) | 0.67 (0.08) | 0.44 (0.01) |

**Table S3 continued.** Fermentation products (in mmols) and maximum specific growth rate µ_max_ for the strains represented in Figure 2. Values in parentheses represent one standard deviation (n ≥ 3 for fermentation products, n = 6 for growth rate).

| Strain | Extracellular amino acids (mmol) | | | | | | | | |
| --- | --- | --- | --- | --- | --- | --- | --- | --- | --- |
|  | Ala | Arg | Asp | Cys | Glu | Gly | His | Ile | Leu |
| MTC | 0.17 (0.03) | 0.00 (0.00) | 0.00 (0.00) | 0.00 (0.00) | 0.00 (0.00) | 0.00 (0.00) | 0.00 (0.00) | 0.00 (0.00) | 0.00 (0.00) |
| LL1004 | 0.02 (0.01) | 0.00 (0.00) | 0.00 (0.00) | 0.00 (0.01) | 0.01 (0.01) | 0.00 (0.00) | 0.00 (0.00) | 0.01 (0.00) | 0.00 (0.00) |
| AG929 | 0.03 (0.01) | 0.00 (0.00) | 0.00 (0.00) | 0.01 (0.01) | 0.02 (0.02) | 0.00 (0.00) | 0.00 (0.00) | 0.01 (0.00) | 0.00 (0.00) |
| LL1319 | 0.03 (0.00) | 0.00 (0.00) | 0.00 (0.00) | 0.01 (0.01) | 0.01 (0.01) | 0.00 (0.00) | 0.00 (0.00) | 0.01 (0.00) | 0.00 (0.00) |
| LL1565 | 0.03 (0.00) | 0.00 (0.00) | 0.00 (0.00) | 0.00 (0.00) | 0.02 (0.02) | 0.00 (0.00) | 0.00 (0.00) | 0.01 (0.00) | 0.00 (0.00) |
| LL1391 | 0.04 (0.00) | 0.00 (0.00) | 0.00 (0.00) | 0.00 (0.00) | 0.01 (0.01) | 0.01 (0.01) | 0.00 (0.00) | 0.00 (0.00) | 0.00 (0.00) |
| LL1566 | 0.04 (0.00) | 0.00 (0.00) | 0.00 (0.00) | 0.00 (0.00) | 0.02 (0.02) | 0.00 (0.00) | 0.00 (0.00) | 0.00 (0.00) | 0.00 (0.00) |
| LL1436 | 0.04 (0.01) | 0.00 (0.00) | 0.00 (0.00) | 0.00 (0.00) | 0.01 (0.02) | 0.01 (0.01) | 0.00 (0.00) | 0.00 (0.00) | 0.00 (0.00) |
| LL1437 | 0.03 (0.01) | 0.00 (0.00) | 0.00 (0.00) | 0.00 (0.00) | 0.02 (0.03) | 0.00 (0.01) | 0.03 (0.06) | 0.00 (0.00) | 0.00 (0.00) |
| LL1438 | 0.03 (0.01) | 0.00 (0.00) | 0.00 (0.00) | 0.00 (0.00) | 0.03 (0.03) | 0.01 (0.01) | 0.02 (0.04) | 0.00 (0.00) | 0.00 (0.00) |
| LL1567 | 0.03 (0.01) | 0.00 (0.00) | 0.00 (0.00) | 0.00 (0.00) | 0.01 (0.02) | 0.00 (0.00) | 0.06 (0.09) | 0.00 (0.00) | 0.00 (0.00) |
| LL1568 | 0.02 (0.01) | 0.00 (0.00) | 0.00 (0.00) | 0.00 (0.01) | 0.01 (0.01) | 0.00 (0.00) | 0.04 (0.07) | 0.01 (0.00) | 0.00 (0.00) |
| LL1569 | 0.03 (0.01) | 0.00 (0.00) | 0.00 (0.01) | 0.00 (0.01) | 0.01 (0.02) | 0.00 (0.00) | 0.04 (0.06) | 0.00 (0.00) | 0.00 (0.00) |
| LL1570 | 0.02 (0.00) | 0.00 (0.00) | 0.00 (0.01) | 0.00 (0.01) | 0.02 (0.02) | 0.01 (0.01) | 0.06 (0.08) | 0.01 (0.00) | 0.00 (0.00) |

**Table S4.** Extracellular amino acid measurements (in mmols) for the strains represented in Figure 2. Values in parentheses represent one standard deviation (n ≥ 3).

| Strain | Extracellular amino acids (mmol) | | | | | | | |
| --- | --- | --- | --- | --- | --- | --- | --- | --- |
|  | Lys | Met | Phe | Pro | Ser | Thr | Tyr | Val |
| MTC | 0.00 (0.00) | 0.00 (0.00) | 0.00 (0.00) | 0.00 (0.00) | 0.00 (0.00) | 0.00 (0.00) | 0.00 (0.00) | 0.00 (0.00) |
| LL1004 | 0.00 (0.00) | 0.00 (0.00) | 0.00 (0.00) | 0.01 (0.00) | 0.01 (0.00) | 0.01 (0.00) | 0.00 (0.00) | 0.18 (0.02) |
| AG929 | 0.00 (0.00) | 0.00 (0.00) | 0.00 (0.00) | 0.01 (0.01) | 0.01 (0.01) | 0.01 (0.00) | 0.00 (0.00) | 0.20 (0.02) |
| LL1319 | 0.00 (0.00) | 0.00 (0.00) | 0.00 (0.00) | 0.01 (0.00) | 0.01 (0.01) | 0.00 (0.00) | 0.00 (0.00) | 0.16 (0.01) |
| LL1565 | 0.00 (0.00) | 0.00 (0.00) | 0.00 (0.00) | 0.02 (0.01) | 0.01 (0.01) | 0.00 (0.00) | 0.00 (0.00) | 0.15 (0.02) |
| LL1391 | 0.00 (0.00) | 0.00 (0.00) | 0.00 (0.00) | 0.00 (0.00) | 0.02 (0.01) | 0.00 (0.00) | 0.00 (0.00) | 0.09 (0.02) |
| LL1566 | 0.00 (0.00) | 0.00 (0.00) | 0.00 (0.00) | 0.00 (0.00) | 0.01 (0.01) | 0.00 (0.00) | 0.00 (0.00) | 0.07 (0.00) |
| LL1436 | 0.00 (0.00) | 0.00 (0.00) | 0.00 (0.00) | 0.01 (0.00) | 0.02 (0.01) | 0.00 (0.00) | 0.00 (0.00) | 0.09 (0.02) |
| LL1437 | 0.00 (0.00) | 0.00 (0.00) | 0.00 (0.00) | 0.01 (0.00) | 0.02 (0.01) | 0.00 (0.00) | 0.00 (0.00) | 0.13 (0.02) |
| LL1438 | 0.00 (0.00) | 0.00 (0.00) | 0.00 (0.00) | 0.01 (0.00) | 0.03 (0.02) | 0.00 (0.00) | 0.00 (0.00) | 0.17 (0.01) |
| LL1567 | 0.00 (0.00) | 0.00 (0.00) | 0.00 (0.00) | 0.01 (0.00) | 0.02 (0.01) | 0.00 (0.00) | 0.00 (0.00) | 0.13 (0.03) |
| LL1568 | 0.00 (0.00) | 0.00 (0.00) | 0.00 (0.00) | 0.01 (0.00) | 0.03 (0.01) | 0.00 (0.00) | 0.00 (0.00) | 0.14 (0.02) |
| LL1569 | 0.00 (0.00) | 0.00 (0.00) | 0.00 (0.00) | 0.00 (0.00) | 0.03 (0.02) | 0.00 (0.00) | 0.00 (0.00) | 0.14 (0.02) |
| LL1570 | 0.00 (0.00) | 0.00 (0.00) | 0.00 (0.00) | 0.00 (0.00) | 0.02 (0.01) | 0.00 (0.00) | 0.00 (0.00) | 0.12 (0.02) |

**Table S4 continued.** Extracellular amino acid measurements (in mmols) for the strains represented in Figure 2. Values in parentheses represent one standard deviation (n ≥ 3).

| Strain | Replicate | Ethanol titer (mM) | Avicel consumed (g glucose eq./L) | Ethanol yield  (% theoretical maximum) | Maximum volumetric ethanol productivity  (g L^-1^ h^-1^) |
| --- | --- | --- | --- | --- | --- |
| LL1319 | 1 | 491 | 86.0 | 46 | 0.63 |
|  | 2 | 481 | 87.5 | 45 | 0.69 |
| LL1570 | 1 | 635 | 95.7 | 54 | 0.87 |
|  | 2 | 533 | 83.8 | 52 | 0.81 |
|  | 3 | 489 | 76.4 | 52 | 0.55 |
|  | 4 | 542 | 75.4 | 58 | 0.56 |
|  | 5 | 565 | 83.4 | 55 | 0.78 |
|  | 6 | 543 | 86.7 | 51 | 0.82 |
| P value | | 0.020 | 0.347 | 0.003 | 0.338 |

**Table S5.** Summary of results from high solids (100 g/L Avicel) fermentation. Ethanol titer values are taken from the final time point for each fermentation. Avicel consumed is determined by subtracting residual Avicel concentration at the final time point from the initial Avicel concentration. Replicates 1 and 2 for strains LL1319 and LL1570 are graphically represented in Figure 3; replicates 3 through 6 for strain LL1570 are graphically represented in Figure S3. P-value is based on a comparison of strains LL1319 and LL1570, determined by an unpaired two-tailed Students’ *t*-test. See Additional File 2 for detailed fermentation results.


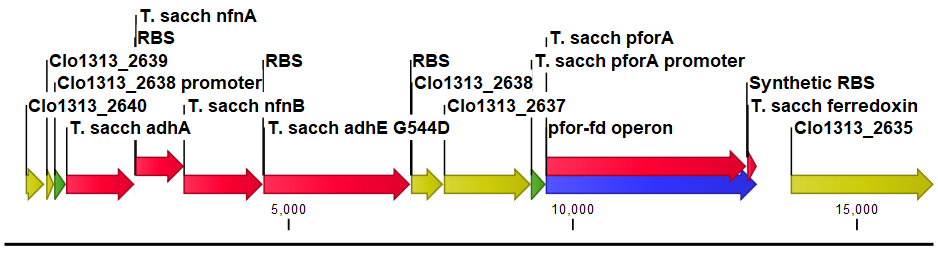


**Figure S1.** Chromosomal location of the *T. saccharolyticum pforA-*ferredoxin operon, using *C. thermocellum* strain LL1391 as an example (the operon uses the *T. saccharolyticum pforA* promoter). Arrows show direction of transcription, with the arrows’ colors indicating if the DNA feature is a native gene (yellow), an introduced *T. saccharolyticum* gene (red), a promoter (green), or the *T. saccharolyticum pforA-*ferredoxin operon (blue). Synthetic ribosome binding sites (flags that read “RBS”) were used to join coding sequences in the designed operon.

**
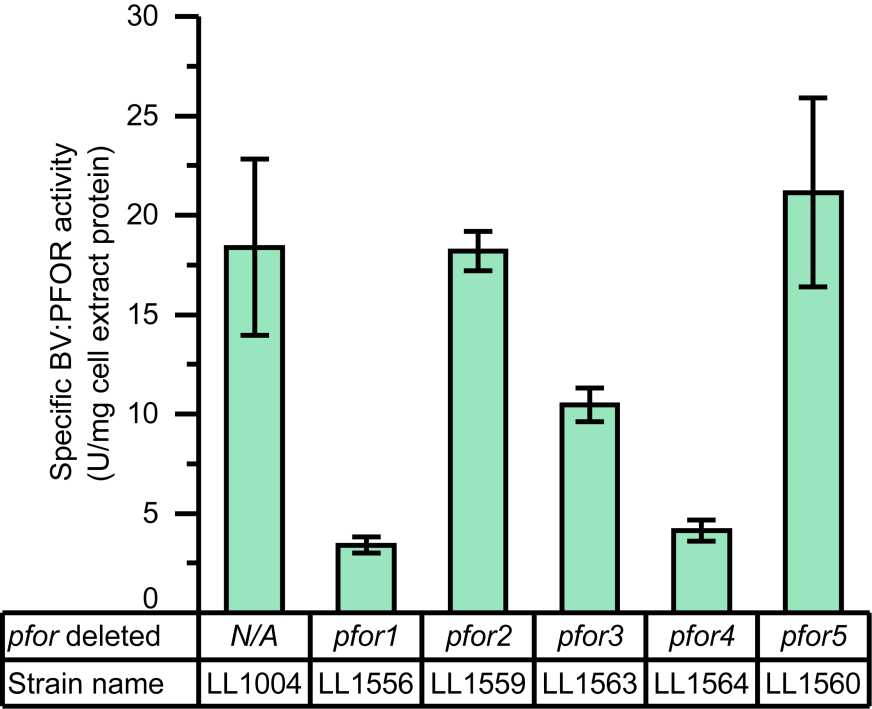
**

**Figure S2.** Specific BV:PFOR activity for wild type *C. thermocellum* (LL1004), and strains of *C. thermocellum* with a deletion of one of the five annotated native *pfors* (see Table 1); all the strains shown here do not contain the *T. saccharolyticum pforA*. For enzyme specific activity, 1 unit (U) is equivalent to the formation of 1 µmol of product per minute. Error bars represent one standard deviation (n ≥ 3).


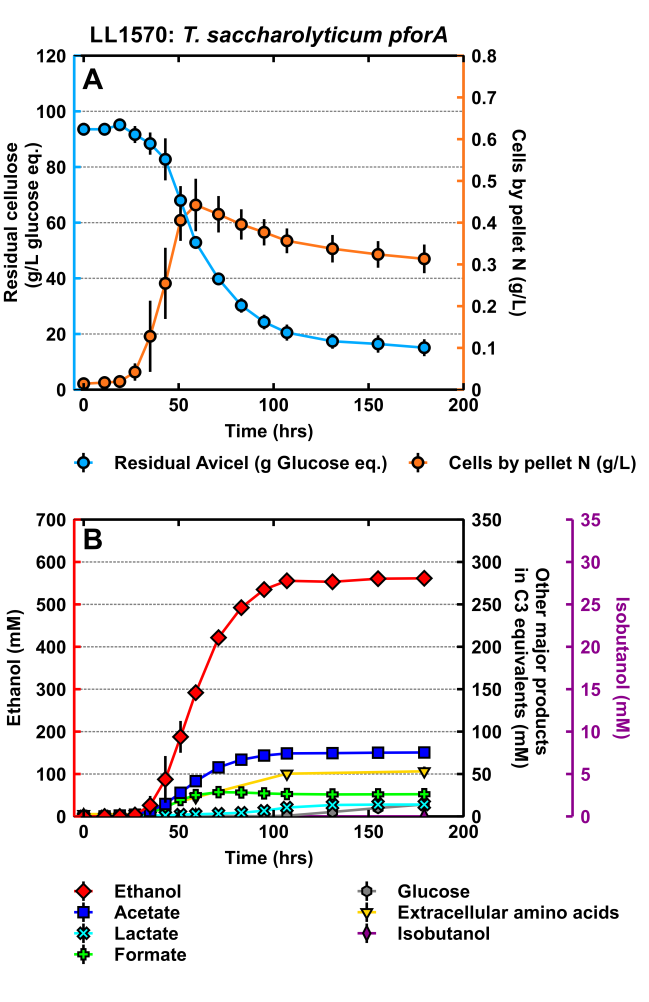


**Figure S3.** Further batch fermentations of strain LL1570. (**A**) Substrate utilization as residual cellulose (light blue) and cells by pellet nitrogen (orange) versus fermentation time. (**B**) Major fermentation products – ethanol (red), acetate (dark blue), lactate (light blue), formate (light green), glucose (gray), isobutanol (purple), and extracellular amino acids (yellow) from the same fermentation runs. Error bars represent mean absolute deviation (n = 4 fermentations). See Additional File 2 for tabular presentation of the data.


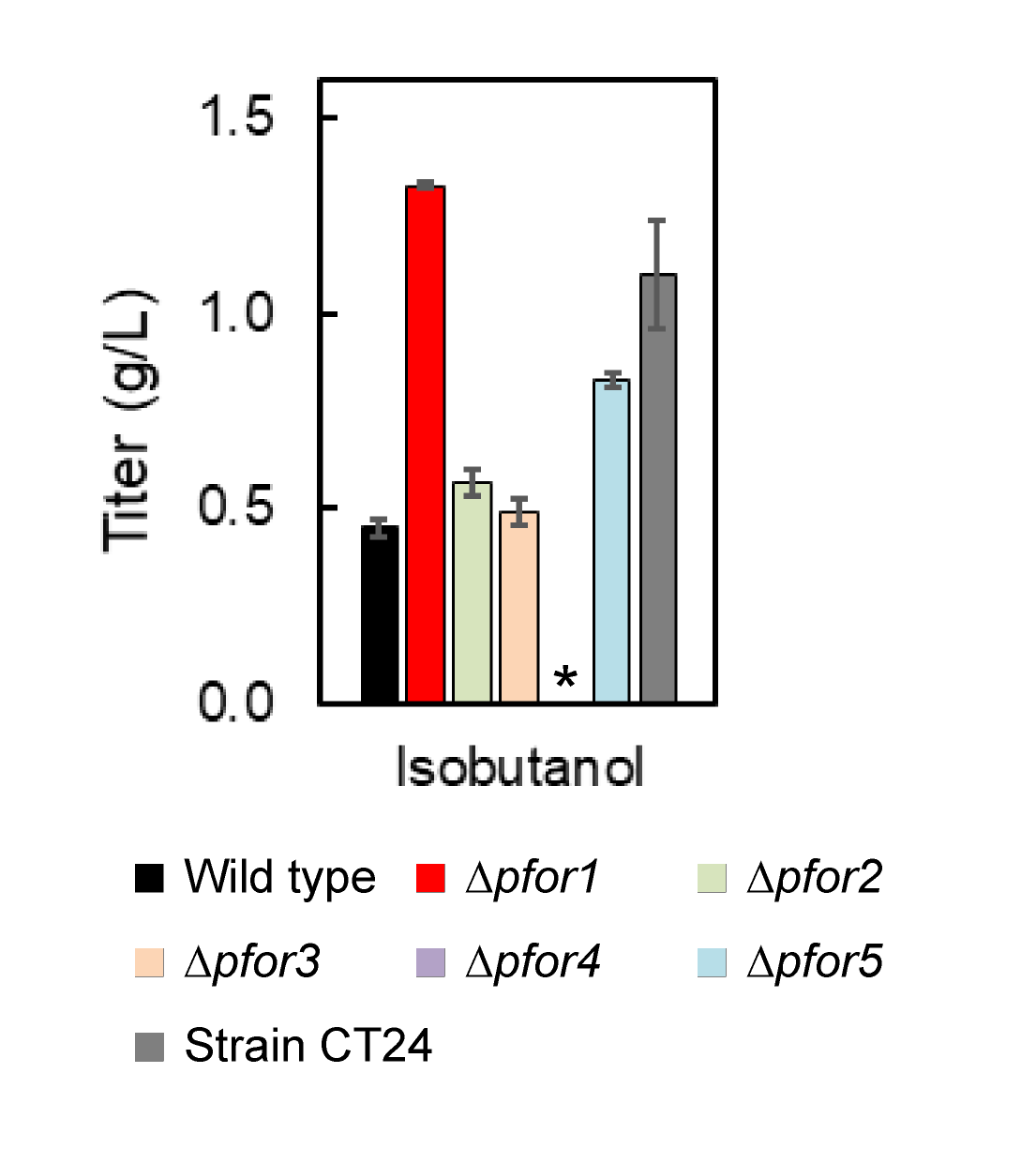


**Figure S4.** Isobutanol production from *C. thermocellum* strains containing a deletion of one of the five annotated *pfor* genes. Strain CT24 is a strain of *C. thermocellum* that was previously engineered for improved isobutanol production . For the strain containing the *pfor4* deletion (indicated by the asterisk in the figure), no isobutanol was detected. Isobutanol production from *C. thermocellum* strains LL1556, LL1559, LL1560, LL1563, and LL1564 were determined as previously described (Lin et al., 2015), and quantified by gas chromatography.
